# Relative Humidity Measurement of Air in Low-Temperature Ranges Using Low-Frequency Acoustic Waves and Correlation Signal Processing Techniques

**DOI:** 10.3390/s22166238

**Published:** 2022-08-19

**Authors:** Miao Guo, Yue Li, Jingmin Gao

**Affiliations:** School of Automation, Beijing Information Science and Technology University, Haidian District, Beijing 100192, China

**Keywords:** acoustic, linear chirp signal, relative humidity, low-frequency sound waves, cross-correlation

## Abstract

Air relative humidity (*RH*) is an important control parameter in many industrial processes. The acoustic method is a novel technique to measure air humidity non-intrusively. Relevant research is limited. Existing methods use ultrasonic waves as a sound source and air humidity is measured by measuring the sound attenuation. In this paper, a novel air humidity measurement system using low-frequency sound waves as a sound source and two acoustic sensors is proposed. Air humidity is acquired by measuring sound speed in the air. Sound speed mainly depends on air temperature, humidity, atmospheric pressure, and air composition. The influence of air temperature, atmospheric pressure, and air constituent concentrations on the *RH* measurement is analyzed theoretically. A 0.1 s linear chirp signal in the frequency range of 200–500 Hz is selected as the sound source. Sound travel time is calculated by cross-correlating the sound signals received by the two acoustic sensors. To improve the accuracy of the sound speed measurement, sound speed under different *RH* points is obtained through reference *RH* experiments and substituted into the calibration equation. Then, equivalent sound path length and systematic delay are estimated using the least squares method. After obtaining these two parameter values, the sound speed measured by the system is closer to the theoretical value at the same *RH* point. In validation experiments using *RH* measured by a thermo-hygrometer as a comparison, the relative errors of the acoustically measured *RH* are within 9.9% in the *RH* range of 40.7–87.1%, and the standard deviation is within 4.8%.

## 1. Introduction

Relative humidity (*RH*) is an important control parameter in many industrial processes which determines the product quality and process economy [1]. Timely and accurate *RH* measurement can help keep the inner environment of warehouses and silos stable, reduce energy consumption, and ensure product quality. The existing *RH* measurement technique is mainly the contact measurement method (humidity-sensitive components). Humidity-sensitive components can be divided into two categories: resistive and capacitive. The characteristic of the humidity-sensitive resistor is that the substrate is covered with a film made of moisture-sensitive material. When the water vapor in the air is adsorbed on the moisture-sensitive film, the resistivity and resistance values of the element change. Then, humidity can be measured. The principle of the humidity-sensitive capacitor is that when the ambient humidity changes, the dielectric constant of the humidity-sensitive capacitor changes, so that the capacitance also changes, and the capacitance change is proportional to the relative humidity. The traditional *RH* sensor has poor linearity and anti-pollution performance. When detecting ambient humidity, the traditional *RH* sensor needs to be exposed to the environment for a long time for it to be measured, and it is easily contaminated which affects its measurement accuracy and long-term stability. The speed of sound in the air mainly depends on the air temperature and *RH*. If the speed of sound in the air and the air temperature can be accurately measured, the *RH* value can be inferred using the relationship between the three parameters. This is the principle of the air *RH* measurement based on the acoustic method proposed in this paper. The acoustic method is a non-invasive measurement technology. Combined with tomography technology, the *RH* distribution information in a two-dimensional area can be obtained [2].

At present, the available sound sources for the measurement of air *RH* by the acoustic method include ultrasonic waves and low-frequency audible sound waves [1,3]. Ultrasonic waves are mainly used for short-distance measurement due to their easy attenuation [3]. Low-frequency sound waves have a smaller attenuation and a longer propagation distance, which have been used to measure indoor temperature, furnace flame temperature, and lake water temperature [4,5,6]. However, the use of low-frequency sound waves to measure air *RH* is relatively limited [1,3]. Therefore, here, an air *RH* measurement method based on low-frequency sound waves and cross-correlation signal processing techniques is proposed.

There are limited studies on the measurement of air *RH* using the acoustic method, and ultrasonic waves are mainly used [1,3]. Motegi et al. proposed an ultrasonic probe device to simultaneously detect air temperature and *RH* through sound velocity and attenuation of sound waves [3]. The experimental results show that the temperature measurement accuracy is high, and the *RH* measurement accuracy needs to be improved. The absolute error of the *RH* measurement result is within 7.53%, and the relative error is within 10.73% (50–90% *RH*). This is mainly because there are many factors that affect the sound wave attenuation, and the attenuation coefficient has a great influence on the *RH* measurement results. Van Schaik et al. proposed a *RH* measurement device that uses a pair of ultrasonic sensors to measure the air flowing in the pipe [1]. The experimental results show that the accuracy is better than 2% above 50 °C. The principle is to measure the speed of sound and air temperature, and then use the relationship between the speed of sound, temperature, and *RH* to infer the *RH*. Their work verifies the feasibility of using the speed of sound to reverse the *RH*, but the coefficients of the calculation formula of the speed of sound need to be calibrated, which needs a high requirement on the calibration device. Sahoo et al. proposed a novel fuzzy-inspired machine learning framework for relative humidity estimation using the time-of-flight of an ultrasonic sensor. Neural networks need to first be trained, and then the trained networks are used to estimate the relative humidity [7]. The authors previously proposed an air temperature measurement device and method based on low-frequency sound waves [8]. The experimental results show that the measurement of air temperature can be achieved. This paper continues the previous research, using low-frequency sound waves to measure air *RH* with sound speed. From the literature review, it can be found that the *RH* measurement accuracy needs to be improved in the low-temperature range. Therefore, this paper focuses on the measurement of air *RH* in low-temperature ranges.

This paper presents the most recent advances in the *RH* measurement of air using low-frequency sound waves and correlation signal processing techniques. The relationship between the speed of sound, temperature, and *RH* is analyzed. The influence of the variation in air temperature, atmospheric pressure, and air composition on the *RH* measurement is investigated through numerical simulation. A measurement system consisting of a low-frequency sound source and two acoustic sensors is constructed. A designed linear frequency sweep signal is sent out through the sound source. Using the cross-correlation algorithm, the propagation time of the sound wave between the acoustic sensors is calculated. Through reference *RH* experiments, the equivalent sound wave propagation path length and systematic delay are obtained, which effectively improves the *RH* measurement accuracy.

The paper is organized as follows: Section 1 is the introduction. Section 2 is the methodology, which includes Section 2.1: relationship between sound speed, air temperature, and *RH*, Section 2.2: the influence of air temperature, atmospheric pressure, and air constituent concentrations on *RH* measurement, Section 2.3: selection of sampling frequency, Section 2.4: sound travel time measurement using chirp signal and cross-correlation algorithm, and Section 2.5: equivalent sound path length and systematic delay. Section 3 introduces the experimental results and discussion, which includes Section 3.1: experimental setup, Section 3.2: sound travel time measurement based on cross-correlation signal processing technique, Section 3.3: estimation of equivalent sound path length and the systematic delay, and Section 3.4: *RH* measurement results. Section 4 provides the conclusions.

## 2. Methodology

**Figure 1:**
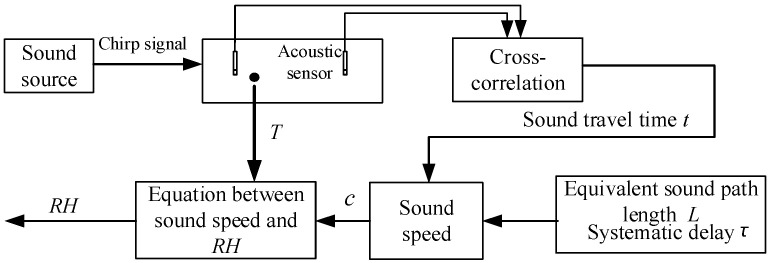
Principle of the air *RH* measurement system based on low-frequency sound waves.

Figure 1 shows the principle of the air *RH* measurement using low-frequency sound waves and acoustic sensors. The sound source, two acoustic sensors, and the sensing head of the reference thermo-hygrometer are fixed at the same height. The sound source sends out the designed low-frequency sound waves, and then two acoustic sensors collect the sound waves. The sound travel time, *t* (s), between the two acoustic sensors is calculated using the cross-correlation algorithm. Sound speed, *c* (m/s), is calculated as:

(1) $$c=\frac{L}{t-\tau }$$

where L is the equivalent sound path length between the two acoustic sensors (m), and τ is the systematic delay (s) [9]. The values of L and τ are acquired through reference *RH* experiments. After calculating sound speed, *c*, the *RH* (%) of air is acquired using the equation between sound speed, air temperature, and *RH*. As sound speed also depends on air temperature, the air temperature, *T* (°C), between the two acoustic sensors needs to be measured using traditional temperature sensors. A detailed introduction about the cross-correlation algorithm and the equivalent sound path length and systematic delay estimation is provided in Section 2.4 and Section 2.5, respectively.

### 2.1. Relationship between Sound Speed, Air Temperature and RH

Sound speed in the air mainly depends on air temperature and *RH*. Sound speed, *c* (m/s) [10], is:

(2) $$c=\sqrt{\frac{\gamma ZR\left(T+273.15\right)}{M_{a}\left[1-x_{w}\left(1-\frac{M_{w}}{M_{a}}\right)\right]}}$$

where γ is the specific heat ratio, *Z* is the compressibility factor, *R* is the universal gas constant (J/(mol·K)), *M_a_* is the molar mass of dry air (kg/mol), *x_w_* is the mole fraction of water vapor, and *M_w_* is the molar mass of water content (kg/mol). *x_w_* is calculated from the measured *RH* as:

(3) $$x_{w}=\frac{0.01RHf_{1}P_{sv}}{P}$$

where *f_1_* is the enhancement factor for water vapor, *P_sv_* is the saturation vapor pressure (Pa), and *P* is the atmospheric pressure (Pa).

**Figure 2:**
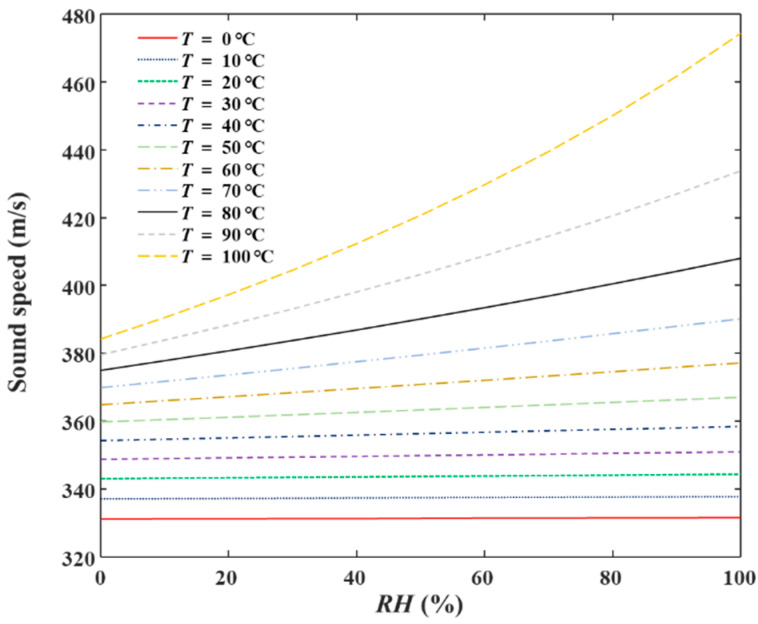
Ideal sound speed as a function of *T* and *RH* (*P* = 102.2 kPa).

*P* in the laboratory where this research was undertaken was 102.2 kPa. Assuming *T* is 0–100 °C, *RH* is 0–100%, and air constituent concentrations are the same as the standard air, then *c* calculated from Equation (2) is as shown in Figure 2. The sound speed has a monotonic relationship with air *RH* for a given air temperature, *T.* With the measured *c*, *T*, *P*, and assuming that air constituent concentrations are the same as that of standard air, *RH* is calculated inversely from Equation (2). From Figure 2 it can be inferred that the influence of *RH* on sound speed is low in the low-temperature range, while the influence is obvious in high-temperature ranges. The accuracy of existing traditional temperature sensors is high. This means that the sound speed measurement accuracy needs to be high enough when the air temperature is low. Therefore, a couple of reference *RH* experiments were conducted to obtain the equivalent sound path length between the two acoustic sensors and the systematic delay.

### 2.2. The Influence of Air Temperature, Atmospheric Pressure and Air Constituent Concentrations on RH Measurement

As air temperature, atmospheric pressure, and air constituent concentrations also affect the sound speed, to increase the measurement accuracy of *RH*, the influence of the three factors on acoustic *RH* measurement results was analyzed theoretically and corresponding technical solutions are provided.

#### 2.2.1. Air Temperature

**Figure 3:**
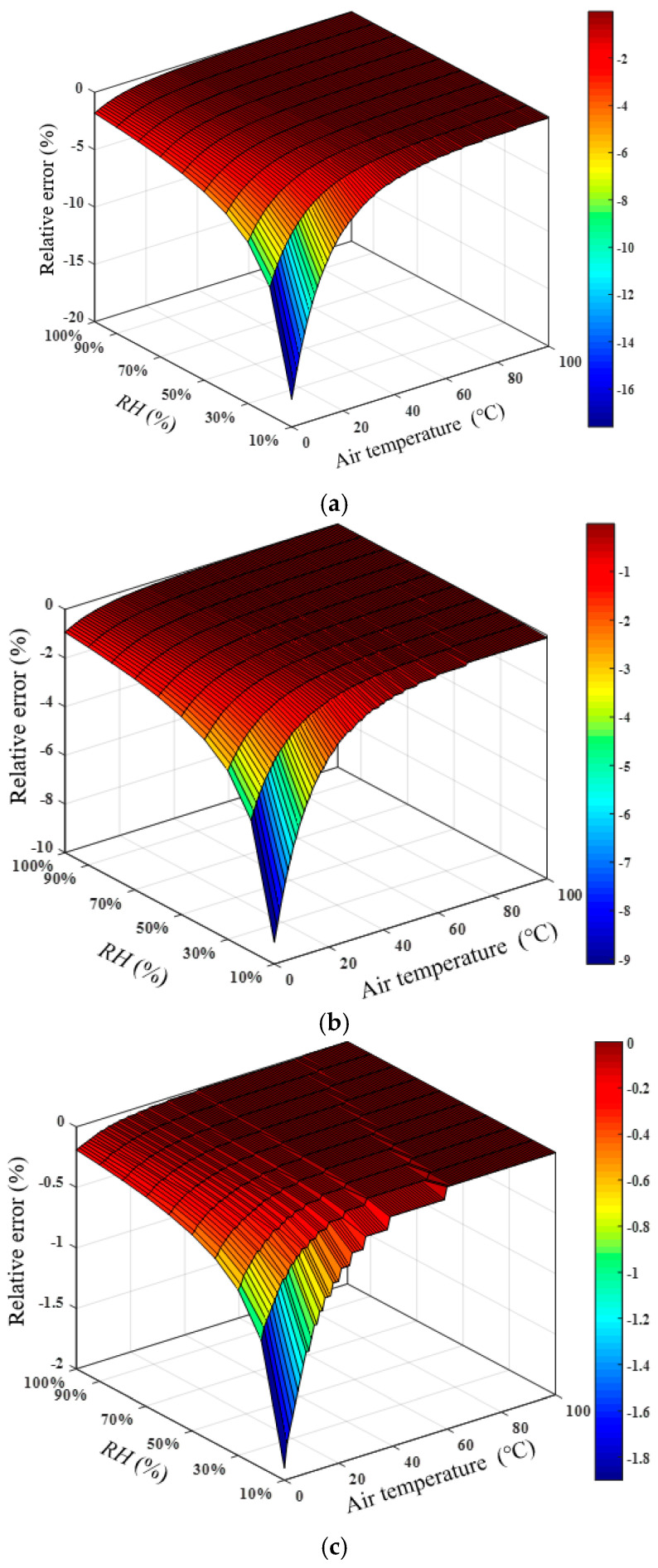
Measurement error of *RH* caused by the variation in *T*. (**a**) The temperature error measured by the thermometer is ±1 °C. (**b**) The temperature error measured by the thermometer is ±0.5 °C. (**c**) The temperature error measured by the thermometer is ±0.1 °C.

In the study of the influence of temperature on the *RH* measurement, it is assumed that the air constituent concentrations and atmospheric pressure are basically unchanged during the measurement process (standard air, atmospheric pressure is 102.2 kPa), which is also in line with the general actual measurement environment. Assuming that actual *RH* is 10–100% (from 10% is used to calculate the relative error of *RH*) and *T* is 0–100 °C, the sound speed can be calculated using Equation (2). Assuming that the actual measured sound speed is equal to the ideal sound speed value, that is, there is no error in the sound speed measurement, the temperature error measured by the thermometer was ±1, ±0.5, and ±0.1 °C. *RH* of air can be calculated with the measured *T* and sound speed. Then, the relative errors of the *RH* measurement results caused by the actual temperature measurement error were acquired, which are shown in Figure 3a–c. The relative error of the *RH* measurement caused by temperature fluctuation was relatively low in the high-temperature and high-*RH* area. The relative error was relatively high in the low-temperature and low-humidity area, which is also the limitation of the acoustic humidity measurement. Therefore, to improve the accuracy of the *RH* measurement, it is necessary to ensure the accuracy of the temperature measurement. The accuracy of the reference temperature measurement device used in the experiments can reach ±0.5 °C. More accurate thermocouples or thermal resistances can be used, and the temperature measurement accuracy can also be improved by using multi-point measurement and averaging.

#### 2.2.2. Atmospheric Pressure

**Figure 4:**
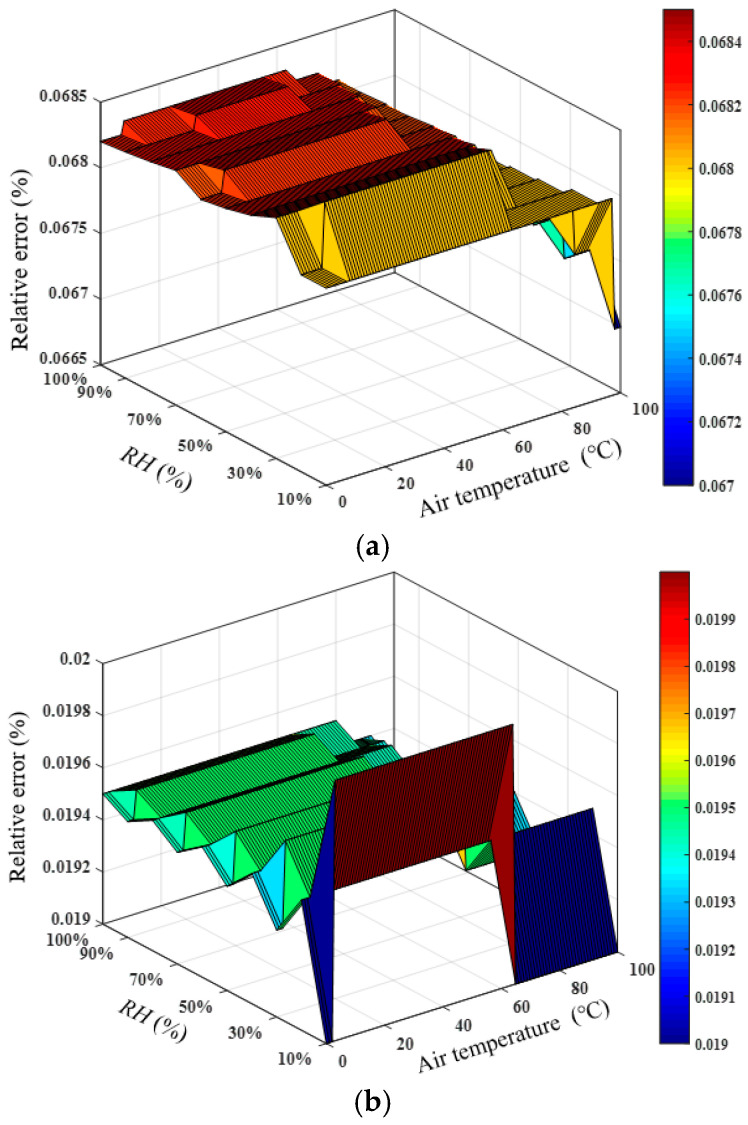
Measurement errors of *RH* caused by the variation in atmospheric pressure. (**a**) P=109.2 kPa and (**b**) P=104.2 kPa.

According to the atmospheric pressure information provided by the National Physical Laboratory in the United Kingdom, the atmospheric pressure change at the same location in a year is within 7 kPa, and the atmospheric pressure change on the same day will not exceed 2 kPa. To analyze the influence of atmospheric pressure fluctuations on the humidity measurement results, assuming that the actual atmospheric pressure is 102.2 kPa, the air temperature is 0–100 °C, and the *RH* is 10–100%, the actual speed of sound can be calculated. Assuming that the atmospheric pressure used in the acoustic temperature measurement is 109.2 and 104.2 kPa, the relative humidity of the air can be calculated according to the actual sound speed, temperature, and atmospheric pressure, and then the relative humidity measurement error caused by the change of atmospheric pressure can be obtained, as shown in Figure 4. The fluctuation of atmospheric pressure within a year will cause the relative *RH* measurement error within 0.07%, and the relative *RH* measurement error caused by the fluctuation of atmospheric pressure in one day was less than 0.02%, which indicates that the atmospheric pressure fluctuation has little influence on the relative humidity measurement. Therefore, when calculating the relative humidity of the air, the atmospheric pressure of the day can be assumed to be constant, and the atmospheric pressure value at a certain time of the day is adopted.

#### 2.2.3. Air Constituent Concentrations

Variations in air constituent concentrations will inevitably affect the sound speed in the air. The study of the quantitative influence of the air composition on *RH* measurement results puts forward high requirements of hardware equipment. The current experimental conditions are difficult to achieve. However, in the general actual measurement environment, the air composition at the same location is relatively consistent, and there will be no major air composition changes (that is, the standard air composition). At the same time, in the method of acoustic measurement of *RH* proposed in this paper, the method of reference *RH* experiments is adopted, thereby reducing the influence of the fluctuation of the air composition.

### 2.3. Selection of Sampling Frequency

**Figure 5:**
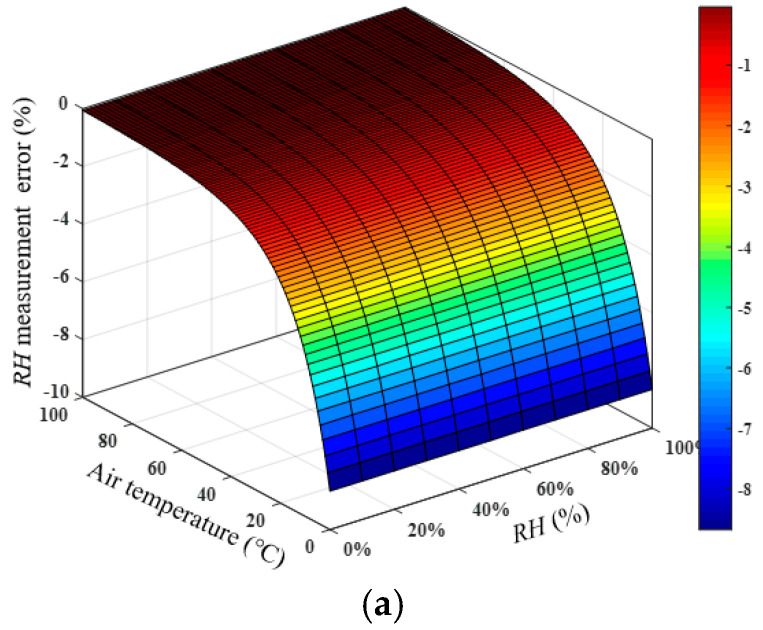

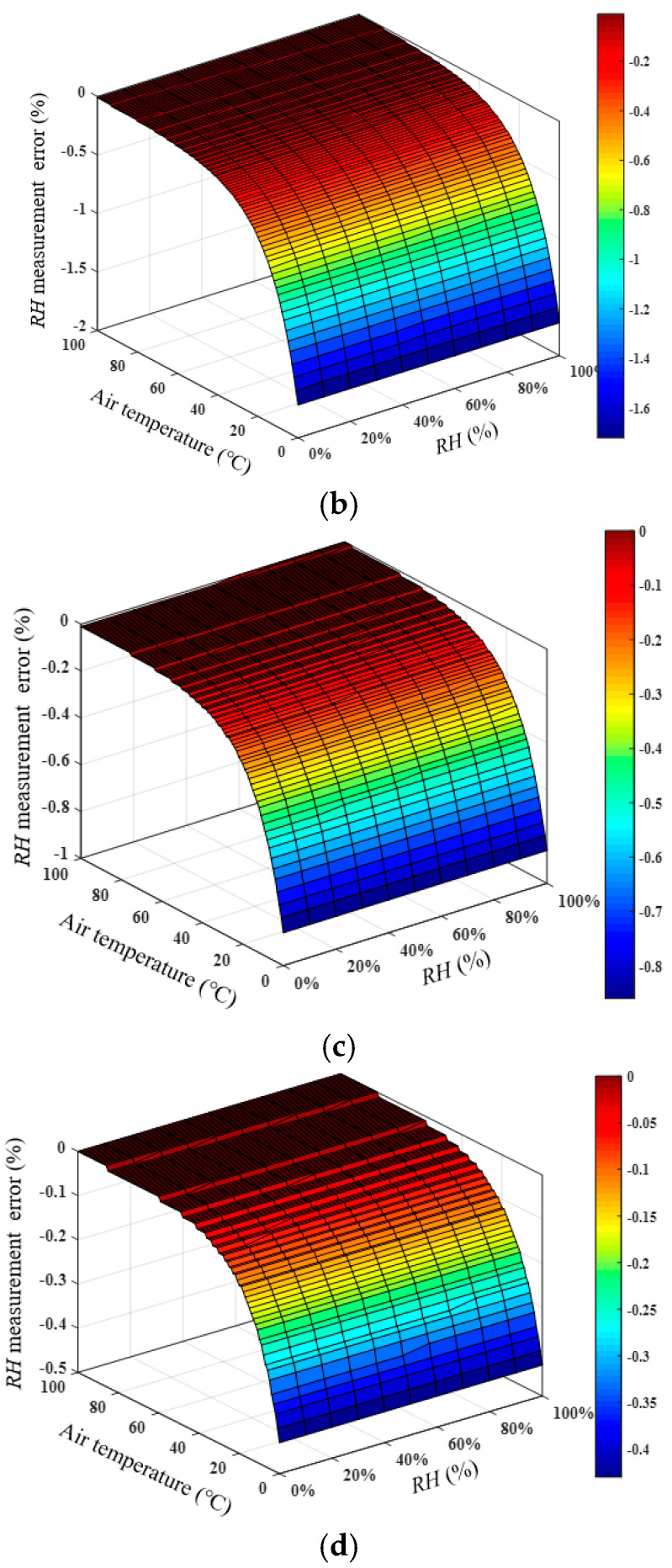
Measurement errors of *RH* caused by the sampling frequency when the distance between the two acoustic sensors was 0.4 m. (**a**) *Fs* = 100 kHz, (**b**) *Fs* = 500 kHz, (**c**) *Fs* = 1 MHz, (**d**) *Fs* = 2 MHz.

**Figure 6:**
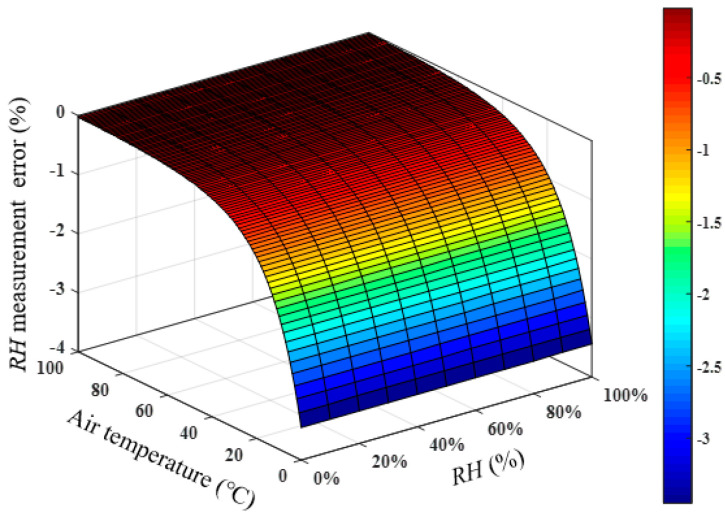
Measurement errors of *RH* caused by the sampling frequency (*Fs* = 100 kHz) when the distance between the two acoustic sensors was 1 m.

The sampling frequency will affect the accuracy of the sound travel time measurement and thus the *RH* measurement results. Assuming that the sampling frequency is *Fs*, then the time resolution is 1/*Fs*. To choose a suitable sampling frequency, the humidity measurement error caused by the time resolution was first theoretically analyzed. During the experiments, the atmospheric pressure, *P*, was 102.2 kPa, and assuming that the air temperature *T* is 0~100 °C, *RH* is 0~100%, and the air composition is consistent with the standard air, the corresponding ideal sound speed can be calculated with Equation (2). The distance between the two acoustic sensors was 0.4 m, and the ideal sound travel time, *t*, was acquired with the sound speed. Assuming that the sound travel time measurement error is only the time resolution error caused by the sampling frequency, thus the actual sound travel time is (*t* + 1/*Fs*) (or *t* − 1/*Fs*, the result is the same). Then, sound speed can be calculated and air humidity *RH*_1_ is derived from Equation (2). Therefore, the *RH* measurement error caused by the sampling frequency is (*RH*_1_ − *RH*). Figure 5a–d show the *RH* measurement error caused by the sampling frequency when *Fs* was 100 kHz, 500 kHz, 1 MHz, and 2 MHz. The *RH* measurement error decreased with the sampling frequency. When *Fs* was lower than 100 kHz, the *RH* measurement error was within 10%, which will seriously affect the *RH* measurement results. The error will be larger, as the sound travel time measurement error does not only come from the sampling frequency. The *RH* measurement error was within 0.5% when the sampling frequency was 2 MHz. Therefore, *Fs* was set to 2 MHz in the experiments. If the distance between the two acoustic sensors was increased, then the *RH* measurement error caused by the sampling frequency decreased. Figure 6 shows the *RH* measurement error when *Fs* was 100 kHz and the distance between the two acoustic sensors was 1 m.

### 2.4. Sound Travel Time Measurement Using Chirp Signal and Cross-Correlation Algorithm

**Figure 7:**
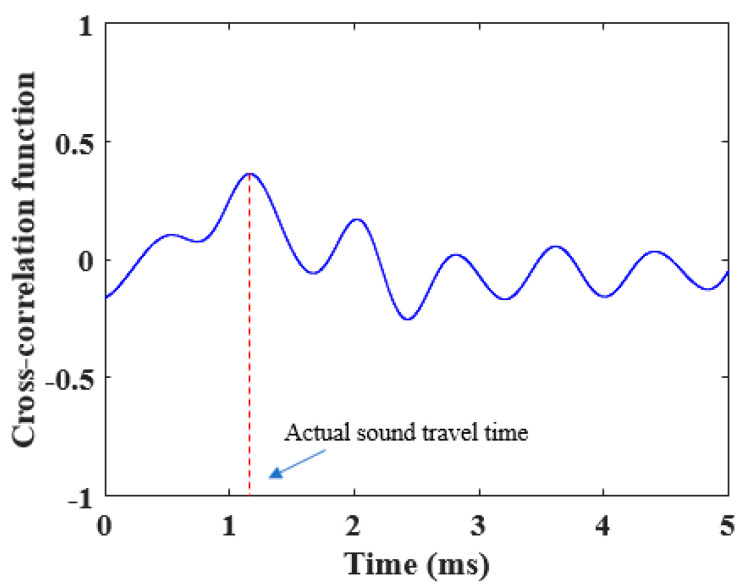
Cross-correlation function of the sound signals from the acoustic sensors 1 and 2.

The chirp signal is widely used as a sound source in the sound travel time measurement [11]. The chirp signal can suppress the side lobe amplitude in the cross-correlation result, which helps to improve the accuracy and stability of the sound travel time measurement. Therefore, the chirp signal was used as the sound source signal in this study. When sound waves emitted by the sound source are received by acoustic sensors 1 and 2, the sound travel time can be calculated through the time difference of the arrival of sound waves. Threshold and peak detection methods based on the amplitude of the received acoustic signal are susceptible to noise and attenuation, resulting in inaccurate temperature measurements. Cross-correlation is a widely used time delay calculation method [12,13,14], and its principle is based on the similarity of the signals received by the sensors. Assuming that the time domain signals $S_{1}\left(t\right)\;$ and $\;S_{2}\left(t\right)$ are the acoustic signals received by the acoustic sensors 1 and 2, the cross-correlation function, $R_{12}\left(m\right)$, between them can be expressed as:

(4) $$R_{12}\left(m\right)=\frac{\sum_{k=1}^{N-M}S_{1}\left(k\right)S_{2}\left(k+m\right)\;}{\sqrt{\sum_{k=1}^{N-M}S_{1}^{2}\left(k\right)}\sqrt{\sum_{k=1}^{N-M}S_{2}^{2}\left(k\right)}},\;0\leq \;m\leq M$$

where $S_{1}\left(k\right)$ and $S_{2}\left(k\right)$ represent the sampled values of signals $S_{1}\left(t\right)\;$ and $\;S_{2}\left(t\right)$, respectively, *N* is the length of the sampled signal for cross-correlation analysis (*N* = 200,000), and *m* is the sampling points of delay (*m* = 0, 1, 2, …, *M*). *M* is the maximum delay point. Figure 7 shows a typical cross-correlation result of sound signals received by acoustic sensors 1 and 2.

The location of the peak point in $R_{12}\left(m\right)$ is the actual delay point, which is the actual sound travel time. Based on the actual measurement of the received sound wave components, there are two overlapping components of reflected sound and direct sound. Therefore, to minimize the impact of reflected sound waves on the cross-correlation results, the maximum delay point, *M*, was set to 10,000 (5 ms), which is larger than the possible actual acoustic travel time.

To increase the amplitude of the main peak in the cross-correlation results and thus the signal-to-noise ratio, oversampling signal processing methods can be applied [15,16]. Oversampling can make up for the lack of hardware sampling frequency and improve the accuracy of acoustic travel time measurements, which ultimately improve the *RH* measurement accuracy.

### 2.5. Equivalent Sound Path Length and Systematic Delay

To improve the sound speed measurement accuracy, Equation (1) was adopted to calculate the sound speed in the air. When using a sound source and two acoustic sensors to measure the speed of sound, the acoustic center of acoustic sensors is not their geometric center. The measured distance between the two acoustic sensors is not the actual sound path length [9]. In addition, due to the difference in the working time of the two acoustic sensors to convert the sound wave signal into an electrical signal and amplify it, there is a systematic delay in the calculation result of the sound wave propagation time. More importantly, the theoretically calculated speed of sound is not exactly equal to the actual speed of sound in a specific measurement environment. To improve the sound velocity measurement accuracy, the equivalent sound path length, *L*, and the systematic delay, *τ*, must be determined by reference *RH* experiments.

Zhou et al. proposed a calibration method for an ultrasonic temperature measurement system [9]. Zhou’s calibration equations were adopted in this paper to calculate the equivalent sound path length, *L*, and the systematic delay, *τ*. Air humidity was set to *m* points and the sound travel time, $t_{RH_{i}}$, was measured ten times under each *RH* point ($RH_{1},\;\ldots ,\;RH_{m}$). The average value of $t_{RH_{i}}$ was substituted into Equation (5). f(T, RH) is the theoretical sound speed under the measured air temperature and *RH*. The equivalent sound path length, *L*, and the systematic delay, *τ*, are the final values when prediction errors $e_{1},\;\ldots ,\;e_{m}$ are minimized.

(5) $$\{\begin{array}{c} \begin{array}{c} t_{RH_{1}}=\frac{L}{f\left(T_{1},\;RH_{1}\right)}+\tau +e_{1}\\ t_{RH_{2}}=\frac{L}{f\left(T_{2},\;RH_{2}\right)}+\tau +e_{2} \end{array}\\ \begin{array}{c} \vdots \\ t_{RH_{m}}=\frac{L}{f\left(T_{m},\;RH_{m}\right)}+\tau +e_{m} \end{array} \end{array}$$

For Equation (5), a least squares optimization algorithm can be used to estimate the two objective parameters in the linear equations. Then, the equivalent sound path length, $\hat{L}$, and the systematic delay, $\hat{\tau }$, can be calculated with Equations (6) and (7):

(6) $$\left[\begin{array}{c} \hat{L}\\ \hat{\tau } \end{array}\right]={\left(A^{T}A\right)}^{-1}A^{T}X$$

(7) $$A=\left[\begin{array}{cc} \begin{array}{c} 1/f\left(T_{1},\;RH_{1}\right)\\ \begin{array}{c} 1/f\left(T_{2},\;RH_{2}\right)\\ \begin{array}{c} \vdots \\ 1/f\left(T_{m},\;RH_{m}\right) \end{array} \end{array} \end{array} & \begin{array}{c} 1\\ \begin{array}{c} 1\\ \begin{array}{c} 1\\ 1 \end{array} \end{array} \end{array} \end{array}\right],\;X=\left[\begin{array}{c} t_{RH_{1}}\\ \begin{array}{c} t_{RH_{2}}\\ \begin{array}{c} \vdots \\ t_{RH_{m}} \end{array} \end{array} \end{array}\right]$$

## 3. Experimental Results and Discussion

### 3.1. Experimental Setup

**Figure 8:**
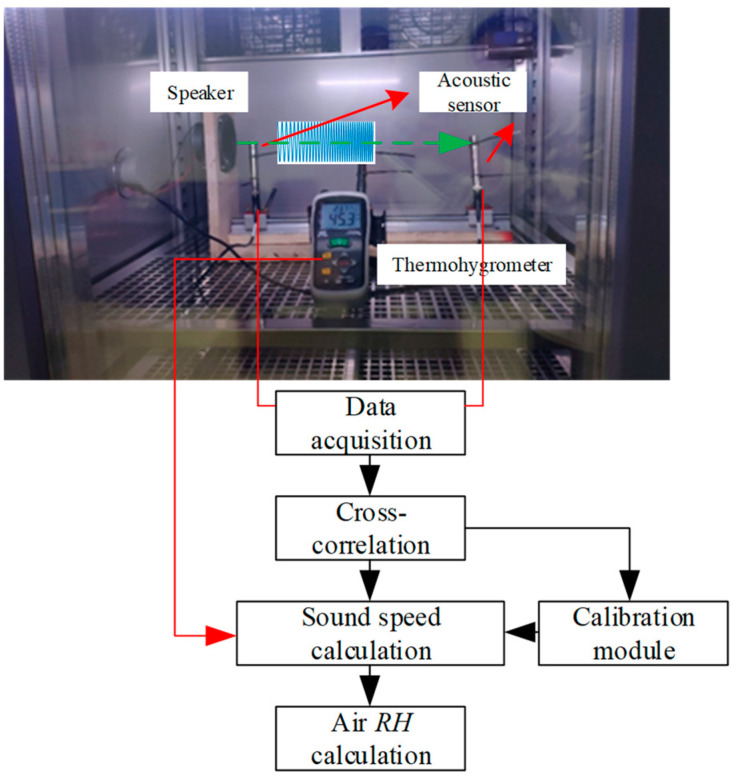
Experimental setup of the air *RH* measurement system.

Figure 8 shows the experimental setup of the air *RH* measurement system using the acoustic method. The sound signal was generated by the computer and sent out by the loudspeaker after being amplified by the sound card and power amplifier. The loudspeaker is rated at 30 W, has an impedance of 8 Ω, and operates from 60 Hz to 20 kHz. The center of the loudspeaker and the sensing element of the acoustic sensors were at the same height, and the loudspeaker, the acoustic sensors, and the thermo-hygrometer were on the same straight line. Two acoustic sensors (Model type MPA201, BSWA TECHNOLOGY Co., Ltd., Beijing, China) were used to collect the sound signals. The sensitivities of acoustic sensors 1 and 2 were 42.2 and 42.1 mV/Pa, respectively. The position of the two acoustic sensors was fixed and the distance between the two acoustic sensors was 0.4 m. A data acquisition card (NI, USB-6366, National Instruments, Austin, TX, USA) was used to collect the output signals of acoustic sensors synchronously, and its sampling frequency was set to 2 MHz. Air temperature on the path between the two acoustic sensors was measured by a CEM DT-625 thermo-hygrometer (Shenzhen Huashengchang Technology Industry Co., Ltd., Shenzhen, China) (temperature measurement accuracy: ±0.5 °C (−30–100 °C)). To acquire the *RH* measurement error of the acoustic method, the reference *RH* value was also measured using the CEM thermo-hygrometer (range: 0–100%, accuracy: ±2.0% *RH* (0–100%)). The constant temperature and humidity chamber (ETE-GDJS-500L) made by Wuxi Suoyate Testing Equipment Co., Ltd. (Wuxi, China) was used to control the air temperature and humidity. The air temperature range was 10–40 °C, uniformity: ≤2.0 °C (no load), temperature fluctuation within 0.5 °C (no load), and the *RH* range was 30~98%, humidity fluctuation: ±2.5% *RH*.

### 3.2. Sound Travel Time Measurement Based on Cross-Correlation Signal Processing Technique

**Figure 9:**
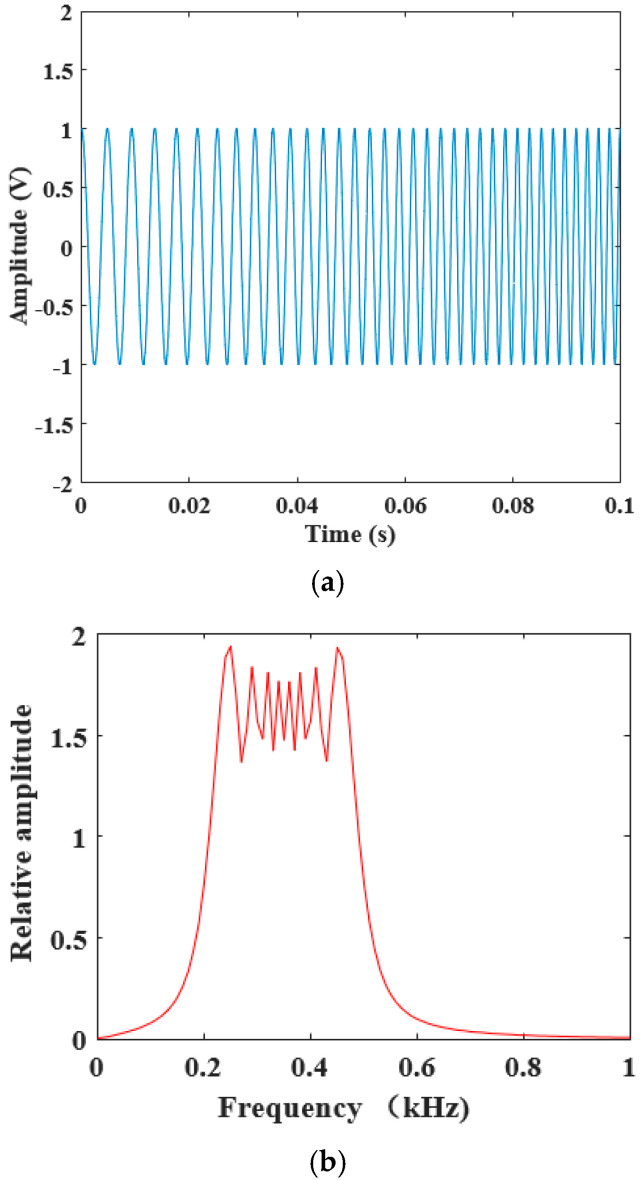
Time domain waveform and frequency spectrum of the ideal linear chirp signal. (**a**) Time domain waveform. (**b**) Frequency spectrum.

**Figure 10:**
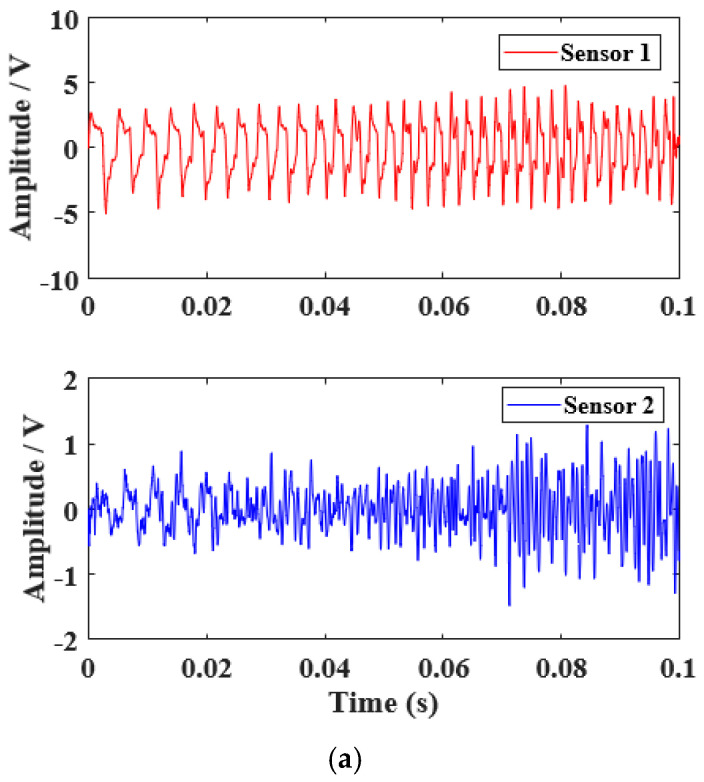

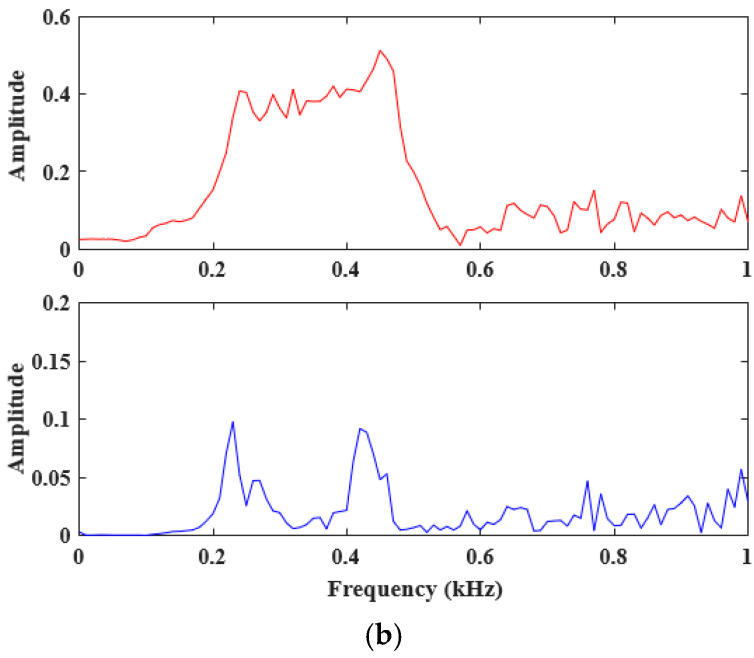
Time domain waveforms and frequency spectra of the signals from the acoustic sensors. (**a**) Time domain waveforms. (**b**) Frequency spectra.

A linear chirp signal with a duration of 0.1 s in the frequency band of 200–500 Hz was chosen as the sound source. Figure 9 shows the waveforms of ideal chirp signals in the time and frequency domains. Figure 10 shows the waveforms of chirp signals received by acoustic sensors in the time and frequency domains. After the sound wave propagated for 0.4 m in the box, the signals received by the acoustic sensors 1 and 2 were still very similar in the time and frequency domains, which is very important to improve the measurement accuracy and stability of the sound wave travel time. Although the acoustic field generated by the speaker was not an ideal far-field configuration, the sound travel time calculated using the cross-correlation method was not affected by the phase difference as it is based on the similarity of the waveforms of the received acoustic signals. The amplitude of the waveforms of acoustic signals is determined by the real part and the imaginary part of the signals. The amplitude frequency response of the two acoustic sensors was consistent through calibration before leaving the factory. The constant temperature and humidity chamber is a limited space which will cause reflection in acoustic waves and change the phase difference of received acoustic waves. Therefore, the sound travel time measurement using the phase difference method was not used in this measurement.

**Figure 11:**
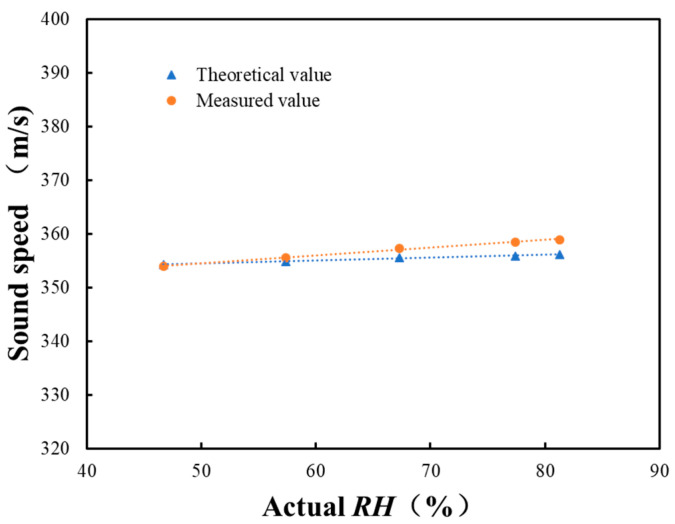
Measured and theoretical values of sound speed (Equation (2)) and their respective fitted curves.

The constant temperature and humidity chamber can adjust the air inside to different temperature and humidity points. The specific temperature and corresponding *RH* are shown in Table 1. Measurements were repeated 10 times under each *RH* point. The temperature data values were the average values of measured temperatures by the CEM DT-625 thermo-hygrometer. The average values and standard deviations (STD) of the sound travel time are listed in Table 1. From Table 1, with the increase of *RH*, sound travel time decreased, which indicates the increase of the sound speed. This complies with the relationship shown in Figure 2. Assuming that the distance between acoustic sensors (0.4 m) is the sound path length and there is no systematic delay, then the sound speed can be measured with the sound travel time in Table 1. The ideal sound speed at each temperature and *RH* point can be acquired with Equation (2). Figure 11 shows the measured and the theoretical values of sound velocity at each humidity point. The measured value deviates from the theoretical value. One reason is the error in the actual sound path length, which was measured by a tape measure. Another reason is the theoretical sound speed is the ideal value. Therefore, the equivalent sound path length, *L*, and the systematic delay, *τ*, need to be calculated through reference *RH* experiments, and then sound speed can be measured more accurately with Equation (1).

### 3.3. Estimation of Equivalent Sound Path Length and the Systematic Delay

**Figure 12:**
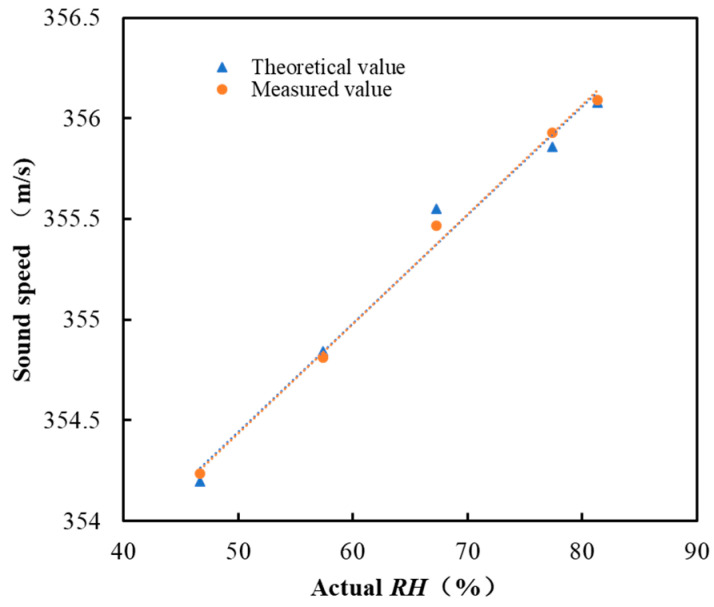
Measured sound speed after reference *RH* experiments and the corresponding theoretical sound speed.

By substituting *T*, *RH*, sound travel time, and the ideal sound speed into Equation (5), the equivalent sound path length, $\hat{L},\;$ and the systematic delay, $\hat{\tau }$, can be calculated with Equations (6) and (7), which are shown in Table 2. Sound speed can be measured with Equation (1), as shown in Figure 12. Comparing Figure 11 and Figure 12, the sound speed measured by the acoustic system is closer to the theoretical value under the same temperature and *RH*. Once the equivalent sound path length and systematic delay are obtained through the reference *RH* experiments, the *RH* of air can be calculated by simply measuring the sound travel time from acoustic sensor 1 to sensor 2. Note that in Table 2, the estimated equivalent sound path length is larger than the actual path length and the systematic delay is larger than the measured sound travel time. This is caused by many factors, such as the fluctuation and uniformity of air *T* and *RH* in the constant chamber. The theoretical sound speed calculation equation was not verified through experiments. Besides, the decay and reverberation of sound waves in the chamber will also affect the accuracy of the sound travel time estimation, which can also be seen in the main peak in Figure 7. All these factors will lead to the deviation of $\hat{L}$ and $\hat{\tau }$ from the actual value. However, the work in this paper focuses on the method itself. $\hat{L}$ and $\hat{\tau }$ in Equation (1) are used to increase the accuracy of the measured sound speed.

### 3.4. RH Measurement Results

**Figure 13:**
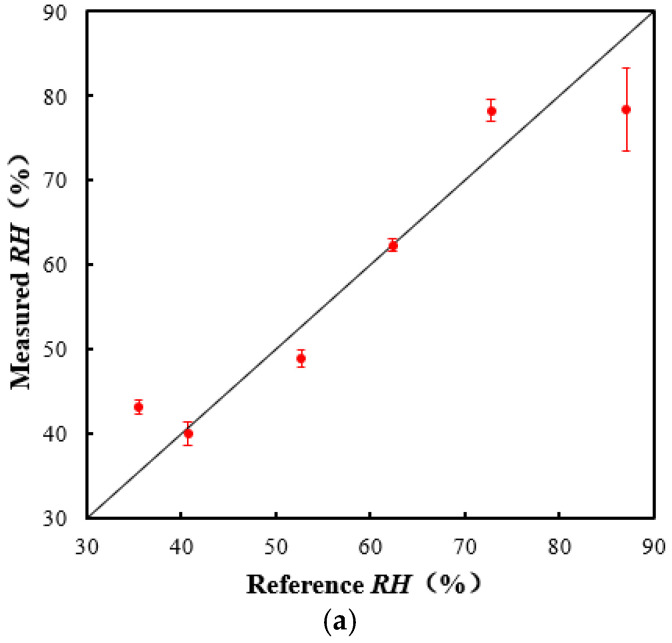

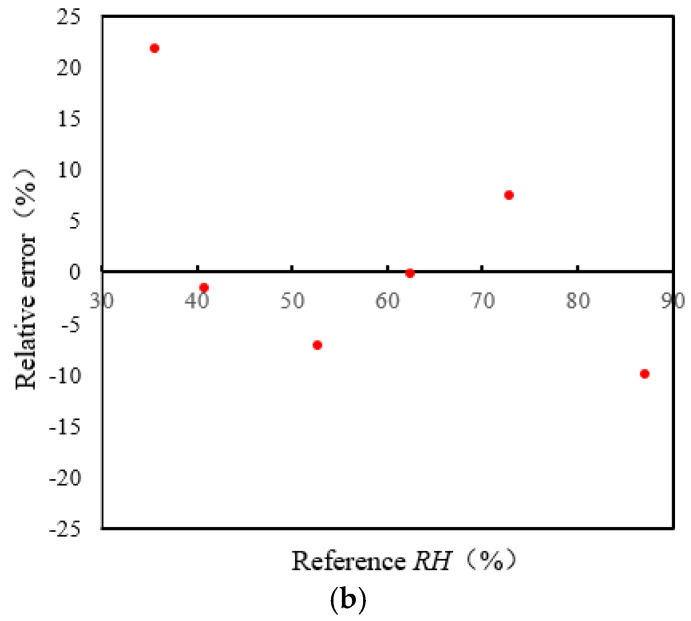
Temperature measurement results versus the reference values. (**a**) Comparison of the measured temperature with reference values. (**b**) Relative error.

To validate the acoustic method, air in the chamber was set to another 8 *RH* points, which were 35.5%, 40.7%, 52.7%, 62.4%, 72.8%, and 87.1%. The corresponding air temperature was measured to be 38.3, 37, 37.1, 37.6, 37.8, and 37.6 °C. Measurements were repeated 10 times at each *RH* point. Figure 13a, b show the average values and relative errors of the measured *RH*. The abscissa is the actual air humidity measured by the thermo-hygrometer. The standard deviation of the measurement results was within 4.8%. The relative error of the measured values was within 9.9% in the *RH* range of 40.7–87.1%. The error was larger when the humidity was low, and the error was less than 10% when the humidity was high. This is also in line with the previous theoretical analysis in Section 2.1. In the low-temperature and low-humidity area, the humidity measurement had high requirements on the accuracy of the sound speed measurement. However, the sound speed measurement was affected by many factors, and thus the measurement error was larger. The error can be decreased by increasing the actual sound path length and the accuracy of the measured sound travel time.

The measurement time of the acoustic *RH* measurement system mainly depends on two factors. One is the time required to measure the sound travel time. For example, a 0.1 s chirp sound wave signal was generated first, and after detecting that the sound wave reached acoustic sensor 1, the sound signal with a length of 0.05 s was collected and cross-correlated. Therefore, the time required was about 0.15 s. Another aspect is the time it takes to measure the air temperature, so using a more responsive thermometer can reduce the humidity measurement time.

To increase the SNR of the received sound signals and the accuracy of the sound travel time measurement, further research will focus on the SNR analysis and more signal processing techniques, such as filtering, oversampling, curve fitting, and multiple cross-correlation. Besides, measurements using three acoustic sensors will be conducted in the future to improve the measurement results.

Due to the performance limitation of the constant temperature and humidity chamber, the air temperature in the chamber can only be set to 10–40 °C. Therefore, *RH* measurements were conducted in the low air temperature range. More experiments need to be conducted in the future to verify the accuracy of the proposed acoustic method. To apply this research in practice, FPGA is essential as high-speed simultaneous sampling is needed. High-precision temperature sensors need to be integrated into the system to provide air temperature information. The hardware system contains a speaker, two acoustic sensors, and a display module of the measured *RH*. The cross-correlation algorithm and the *RH* calculation algorithm are embedded in the system. The measurement system needs to be calibrated in a constant temperature and humidity chamber in advance.

## 4. Conclusions

1. In this study, a method of air *RH* measurement in the low-temperature range using low-frequency sound waves and cross-correlation signal processing techniques was proposed. By cross-correlating the chirp sound signals received by the two acoustic sensors, the sound travel time was acquired. Then, *RH* can be calculated with the equation between sound speed and air humidity.
2. To increase the accuracy of the sound speed measurement, the equivalent sound path length and the systematic delay were calculated from reference *RH* experiments.
3. Comparing with the *RH* measurement results of the thermo-hygrometer, the relative errors of the acoustically measured *RH* were within 9.9% in the *RH* range of 40.7–87.1%, and the standard deviation was within 4.8%. This indicates that low-frequency sound waves can be used to measure air humidity accurately and non-intrusively. The measurement error was larger when the actual humidity was low, and the error was less than 10% when the humidity was high. In the low-temperature and low-humidity area, the humidity measurement had high requirements on the accuracy of the sound speed measurement.
4. The relative error of the *RH* measurement caused by temperature fluctuation was relatively high in the low-temperature and low-humidity area, which is also the limitation of the acoustic humidity measurement. Therefore, to improve the accuracy of the *RH* measurement, it is necessary to ensure the accuracy of the temperature measurement. More accurate thermocouples or thermal resistances can be used, and the temperature measurement accuracy can also be improved by using multi-point measurement and averaging.
5. The atmospheric pressure fluctuation had little influence on the relative humidity measurement. The *RH* measurement error decreased with the sampling frequency.

## Figures and Tables

**Table 1:** Sound travel time under different air temperatures and *RH* values.

T (°C)	*RH* (%)	Sound Travel Time (ms)	STD (μs)
36.9	46.7	1.1300	0.58
37.3	57.4	1.1252	0.71
37.8	67.3	1.1197	0.24
37.7	77.4	1.1158	0.26
37.8	81.3	1.1145	0.76

**Table 2:** Equivalent sound path length and systematic delay estimation.

T (°C)	*RH* (%)	f(t, RH) (m/s)	t (ms)	$\hat{L}\;(m)$	$\hat{\tau }\;(ms)$
36.9	46.7	354.1973	1.1300	1.0570	−1.8539
37.3	57.4	354.8442	1.1252		
37.8	67.3	355.5507	1.1197		
37.7	77.4	355.8598	1.1158		
37.8	81.3	356.0786	1.1145		
